# Modulatory Effect of 2-(4-Hydroxyphenyl)amino-1,4-naphthoquinone on Endothelial Vasodilation in Rat Aorta

**DOI:** 10.1155/2016/3939540

**Published:** 2016-09-08

**Authors:** Javier Palacios, Fredi Cifuentes, Jaime A. Valderrama, Julio Benites, David Ríos, Constanza González, Mario Chiong, Benjamín Cartes-Saavedra, Carlos Lafourcade, Ursula Wyneken, Pamela González, Gareth I. Owen, Fabián Pardo, Luis Sobrevia, Pedro Buc Calderon

**Affiliations:** ^1^Departamento de Ciencias Químicas y Farmacéuticas, Facultad de Ciencias de la Salud, Universidad Arturo Prat, Iquique, Chile; ^2^Experimental Physiology Laboratory (EPhyL), Instituto Antofagasta, Universidad de Antofagasta, Antofagasta, Chile; ^3^Advanced Center for Chronic Diseases, Faculty of Chemical and Pharmaceutical Sciences, University of Chile, Santiago, Chile; ^4^Laboratorio de Neurociencias, Centro de Investigaciones Biomédicas, Universidad de los Andes, Monseñor Alvaro del Portillo 12.455, 7550000 Santiago, Chile; ^5^Facultad de Ciencias Biológicas y Centro UC Investigación en Oncología (CITO), Pontificia Universidad Católica de Chile, Chile; ^6^Cellular and Molecular Physiology Laboratory (CMPL), Division of Obstetrics and Gynecology, School of Medicine, Faculty of Medicine, Pontificia Universidad Católica de Chile, 8330024 Santiago, Chile; ^7^Department of Physiology, Faculty of Pharmacy, Universidad de Sevilla, 41012 Seville, Spain; ^8^University of Queensland Centre for Clinical Research (UQCCR), Faculty of Medicine and Biomedical Sciences, University of Queensland, Herston, QLD 4029, Australia; ^9^Toxicology and Cancer Biology Research Group, Université catholique de Louvain, Brussels, Belgium

## Abstract

The vascular endothelium plays an essential role in the control of the blood flow. Pharmacological agents like quinone (menadione) at various doses modulate this process in a variety of ways. In this study,** Q7**, a 2-phenylamino-1,4-naphthoquinone derivative, significantly increased oxidative stress and induced vascular dysfunction at concentrations that were not cytotoxic to endothelial or vascular smooth muscle cells.** Q7** reduced nitric oxide (NO) levels and endothelial vasodilation to acetylcholine in rat aorta. It also blunted the calcium release from intracellular stores by increasing the phenylephrine-induced vasoconstriction when CaCl_2_ was added to a calcium-free medium but did not affect the influx of calcium from extracellular space.** Q7 **increased the vasoconstriction to BaCl_2_ (10^−3^ M), an inward rectifying K^+^ channels blocker, and blocked the vasodilation to KCl (10^−2^ M) in aortic rings precontracted with BaCl_2_. This was recovered with sodium nitroprusside (10^−8^ M), a NO donor. In conclusion,** Q7** induced vasoconstriction was through a modulation of cellular mechanisms involving calcium fluxes through K^+^ channels, and oxidative stress induced endothelium damage. These findings contribute to the characterization of new quinone derivatives with low cytotoxicity able to pharmacologically modulate vasodilation.

## 1. Introduction

A functional vascular endothelium could have major clinical implications in pathologies such as cardiovascular disease, diabetes, and cancer [1, 2]. Previous studies have shown that the treatment with quinone-related compounds (i.e., 1,4-naphthoquinone derivatives) can impair [3, 4] or improve vascular functions [5]. The underlying mechanism(s) of quinone on vascular functions are still not fully understood. For instance, the observed decreased relaxation and increased contraction of blood vessels induced by menadione was partly explained by inhibition of NO pathway via the formation of reactive oxygen species (ROS). The hypotension and vasorelaxation effects of naphthoquinone-oxime were suggested to be due to activation of soluble guanylate cyclase (sGC), K^+^ channels, and via NO pathway [6].

Quinones are widely distributed molecules in nature, and they are found as endogenous compounds of biological importance (i.e., coenzyme Q in mitochondrial electron chain; vitamin K_2_ in blood coagulation) in humans. Numerous therapeutic drugs, in particular antitumor compounds, are quinone-bearing molecules including anthracyclines (doxorubicin, mitoxanthrone, and daunarubicin), benzoquinones (mitomycin C, geldanamycin), orthonaphthoquinones like *β*-lapachone, and several synthetic compounds which are currently under clinical trials using 1,4-naphthoquinone as pharmacophore group [6–9]. They are reported to cause cytotoxic effects through varied mechanisms such as DNA intercalation, reductive alkylation of biomolecules, and ROS formation through a redox-cycling reaction [10–12]. Regarding this latter mechanism, quinone reduction by 1 or 2 electrons from NADPH-cytochrome P450 reductase leads to a semiquinone free radical which is oxidized back to the former quinone in the presence of molecular oxygen, while oxygen is reduced to superoxide anion [13]. This redox-cycling leads to the formation of other ROS, such as hydrogen peroxide and hydroxyl radicals [14, 15]. Based on this redox-cycling property, several quinones have been used as therapeutic agents against several diseases and pathologies [16–18]. As such they induced cell death by either apoptosis or necrosis, inhibiting cancer cells growth [19–21]. They also activated a senescence program leading to cell cycle arrest [22, 23].

Our observations were that one arylamino-naphthoquinone derivative, namely,** Q7** [2-(4-hydroxyphenyl)amino-1,4-naphthoquinone], was able to provoke a drop in ATP cell content and inhibit cancer cell proliferation [24]. This inhibition induced cancer cells senescence [10], reduction of DNA damage, and inhibition of* in vivo* tumor growth [11]. It also leads to an inhibition of* in vivo* tumor progression by triggering apoptosis, cell cycle arrest, suppression of HIF-1, and uncoupling glycolytic metabolism [25]. All these effects have been attributed to its ability to generate ROS through quinone redox-cycling. Meanwhile, it has been reported that some naphthoquinone derivatives are potent inhibitors of endothelium-dependent vasodilation via an inhibition of endothelial NOS, possibly by interacting with the reductase domain of the enzyme [4, 26]. Indeed, it has been reported that oxidative stress enhances vascular reactivity, most likely by increasing the formation of ROS and decreasing the availability of nitric oxide [27, 28].

Based on these facts and due to the critical role of vascular functions in different pathologies, we have explored new biological activities of** Q7** as to be used beyond the context of cancer, especially as it interferes with oxidative stress and vascular function (reactivity and endothelium), an integral complex involved in the survival or death of these cancerous cells.

## 2. Materials and Methods

### 2.1. Cell Culture

Human umbilical vein endothelial cells (HUVEC) were isolated by collagenase 0.25 mg/mL, Collagenase Type II from Clostridium histolyticum (Boehringer, Mannheim, Germany) digestion from umbilical cords obtained at birth from pregnancies and cultured (37°C, 5% CO_2_) in 1% gelatin-coated petri dishes up to passage 2 in medium 199 (M199; Life Technologies, Carlsbad, CA, USA) containing 5 mmol/L D-glucose, 10% new born bovine serum, 10% fetal bovine serum (FBS), 3.2 mmol/L L-glutamine, and 100 U/mL penicillin-streptomycin (primary culture medium). A7r5 cells, a vascular smooth muscle cell (VSMC) line originally derived from embryonic rat aorta, were purchased from the American Type Culture Collection (ATCC). They were cultured in Dulbecco's Modified Eagle Medium (DMEM; Invitrogen, NY, USA, Sigma-Aldrich) supplemented with 10% FBS and 2 × 10^−3 ^M pyruvate. Prior to experiments, 80–90% confluent A7r5 cells were serum-starved.

### 2.2. MTS Reduction Assay

Briefly, cells (50–60% confluence) were seeded in 96-well plates. Cells were incubated at 37°C in a humidified 5% CO_2_/95% air mixture. HUVECs and A7r5 vascular smooth cell line were incubated in the absence or in the presence of** Q7** (10^−7^ M, 10^−6^ M, 10^−5^ M, and 10^−4^ M) for 48 h, and cytotoxicity was determined using the MTS [3-(4,5-dimethylthiazol-2-yl)-5-(3carboxymethoxyphenyl)-2-(4-sulfophenyl)-2H-tetrazolium] reduction assay. Cell Titer 96® AQueous One Solution Cell Proliferation Assay, Promega, WI, USA. Cyclophosphamide (10^−4^ M) was used as negative control and 10^−5^ M epirubicin (anthracycline drug used for chemotherapy) as positive control. The MTS and** Q7** were dissolved in vehicle (DMSO) at final concentration less than 0.1%. All the assays were performed in quintupled and in 3 independent experiments. The cytotoxicity was calculated in accordance with the formula: cytotoxicity (%) = (1 − (absorbance sample/absorbance control)) × 100. The absorbance of sample was determined in the absence (vehicle; control) or in the presence of** Q7**. The absorbance was measured with a microplate reader (Infinite 200 PRO; Tecan, Switzerland) at 490 nm.

### 2.3. Animals

Male and female Sprague–Dawley rats (4-5 weeks of age, 120–180 g) from the breeding colony at the Antofagasta University were used for this study. All rats were housed in a temperature-controlled, light-cycled (08:00–20:00 hours) room with* ad libitum* access to drinking water and standard rat chow (Champion, Santiago). The assays were conducted according to the Guide for the Care and Use of Laboratory Animals published by the U.S. National Institutes of Health (NIH Publication revised 2013), and the local animal research committee approved the experimental procedure used in the present study.

### 2.4. Isolation of Aortic Rings

Rats were sacrificed through cervical dislocation. The thoracic aorta was quickly excised and placed in cold (4°C) physiological Krebs-Ringer bicarbonate buffer (KRB) containing (×10^−3^ M) 4.2 KCl, 1.19 KH_2_PO_4_, 120 NaCl, 25 Na_2_HCO_3_, 1.2 MgSO_4_, 1.3 CaCl_2_, and 5 D-glucose (pH 7.4). Rings (3–5 mm and 2–4 mg) were prepared after connective tissue was cleaned out from the aorta, taking special care to avoid endothelial damage. Aortic rings were equilibrated for 40 min at 37°C by constant bubbling with 95% O_2_ and 5% CO_2_.

### 2.5. Vascular Reactivity Experiments

Aortic rings of native animals were incubated (30 min) and perfused acutely with** Q7** (10^−6^ M and 10^−5^ M) in the organ bath. The aortic rings from the same animal were studied in duplicate, using different vasoactive substances (phenylephrine [PE], acetylcholine [ACh], sodium nitroprusside [SNP], KCl). The rings were mounted on two 25-gauge stainless steel wires; the lower one was attached to a stationary glass rod and the upper one was attached to an isometric transducer (Radnoti, Monrovia, California). The transducer was connected to a PowerLab 8/35 (Colorado Springs CO) for continuous recording of vascular tension using the LabChart 8 computer program (ADS Instruments).

After the equilibration period, the aortic rings were stabilized by 2 successive near-maximum contractions with KCl (6 × 10^−2^ M) for 10 min. The passive tension on aorta was 1.0 g, which was determined to be the resting tension for obtaining maximum active tension induced by 6 × 10^−2^ M KCl. Ten min after contraction with phenylephrine (PE; 10^−6^ M), cumulative concentrations of acetylcholine (ACh) were added to the medium (10^−8^ to 10^−5^ M). Similar protocols were repeated with SNP (10^−8^ to 10^−6^ M). To study the role of extracellular calcium, experiments were performed with a calcium-free KRB containing (×10^−3^ M) 1.0 EGTA, 4.2 KCl, 1.19 KH_2_PO_4_, 125 NaCl, 25 Na_2_HCO_3_, 1.2 MgSO_4_, and 5 D-glucose (pH 7.4). The aortic rings were preincubated in a KRB with calcium for 30 min; then the KRB was changed with KRB without calcium for 5 min before PE (10^−6^ M) was added. Five min after contraction with PE (10^−6 ^M), cumulative concentrations of CaCl_2_ (0.1 to 1.0 × 10^−3^ M) were added to the medium. In other experiments the contraction was induced by 10^−3 ^M BaCl_2_ for 10 min and then relaxed with 10^−2 ^M KCl. BaCl_2_ is used because it increases vasoconstriction by blocking of inward rectifying K^+^ channels [29–31], thus depolarizing the plasma membrane.

### 2.6. Lipid Peroxidation

Thiobarbituric acid reactive substances (TBARS) were measured in rat aorta homogenates. Samples of homogenates (500 *μ*L; 10 ± 1.4 mg protein/mL) were incubated with vehicle and** Q7** (10^−6^–10^−4^ M) for 30 min and then centrifuged at 3000 ×g for 20 min at 4°C. A 100 *μ*L aliquot of the supernatant was mixed with 200 *μ*L of 10% trichloroacetic acid (TCA) and 4% butylated hydroxytoluene. The mixture was then centrifuged and 140 *μ*L of supernatant (in duplicate) was mixed with thiobarbituric acid (0.67%) and heated for 1 h at 95°C. After cooling to room temperature, 280 *μ*L of butanol-pyridine (15 : 1) was added. After centrifugation (3000 ×g, 20 min) the absorbance was measured at 532 nm.

### 2.7. Nitrite/Nitrate Assay

The production of NO by a segment of the rat aorta (in native rats) was measured by nitrite accumulation using the Griess reaction method [32]. Aortic rings were incubated in KRB constantly bubbled with 95% O_2_ and 5% CO_2_ for 40 min at 37°C. KRB containing (×10^−3^ M) 4.2 KCl, 1.19 KH_2_PO_4_, 120 NaCl, 25 Na_2_HCO_3_, 1.2 MgSO_4_, 1.3 CaCl_2_, and 5 D-glucose (pH 7.4). Aortic rings were incubated at 37°C for 30 min with saline, ACh (10^−5 ^M) or the combination of ACh (10^−5^ M) and** Q7** (10^−5 ^M) or ACh (10^−5 ^M) plus N_w_-nitro-L-arginine methyl ester (L-NAME; 10^−4 ^M). At the end of incubation, samples were collected and nitrate reduction was carried out with Zn dust for 30 min at room temperature. Total nitrites in each sample were determined by the addition of 1% sulfanilamide, followed by 0.1% N-(1-naphthyl) ethylenediamine (NED) in 5% phosphoric acid. The absorbance was measured with a microplate reader (Infinite 200 PRO; Tecan, Switzerland) at 550 nm. Nitrite concentration was expressed as *μ*M/mg tissue and calculated from a standard curve with sodium nitrite.

### 2.8. L-Citrulline Assay

NOS activity was determined by incubation of HUVECs with 10^−4 ^M L-arginine and 9 × 10^−6^ Ci/mL L-[^3^H]arginine (30 minutes, 37°C) in the absence or presence of 10^−4 ^M L-NAME (a NOS inhibitor). In addition, 10^−5 ^M** Q7** was used. HUVECs were incubated in HEPES buffer containing (×10^−3 ^M) 50 HEPES, 100 NaCl, 5 KCl, 2.5 CaCl_2_, and 1 MgCl_2_ (pH 7.4). The fraction of L-[^3^H]citrulline formation inhibited by L-NAME was considered NOS activity [33]. Digested cells (95% formic acid) were passed through an ion-exchange resin Dowex-50W 5 (50X8-200) and L-[^3^H]citrulline was determined in H_2_O eluate as described [34]. NOS activity was calculated by subtracting L-NAME-insensitive L-citrulline from the total L-citrulline.

### 2.9. Determination of Intracellular Calcium in Culture Cell

A7r5 cells were cultured in 35 mm culture dish for confocal microscopy (ibidi, Germany). The cells were washed with Krebs with calcium containing (×10^−3 ^M) 140 NaCl, 5 KCl, 1 CaCl_2_, 1 MgCl_2_, 10 HEPES, and 5.6 glucose, pH 7.4. Then, they were loaded with 10^−5 ^M Fluo-3-AM for 25 min at 37°C and then were again washed with Krebs without calcium containing (×10^−3 ^M) 145 NaCl, 5 KCl, 1 MgCl_2_, 10 HEPES-Na, and 5.6 glucose, pH 7.4. In Krebs free-calcium 5 × 10^−5 ^M BAPTA-AM was used. The cells were preincubated in Krebs without calcium for 5 min before PE (10^−6 ^M) was added, and then 10^−3 ^M of CaCl_2_ was added to the medium. The intensity of Ca^2+^ fluorescence was measured with a laser scanning confocal microscope (Leica TCS SP8, Concord, ON, Canada) and fluorescence was recorded every 5 seconds. Analysis involved determination of pixels assigned to each cell using Image J software. The average pixel value allocated to each cell was obtained with excitation at 506 nm and corrected for background.

### 2.10. Chemicals

**Figure 1:**
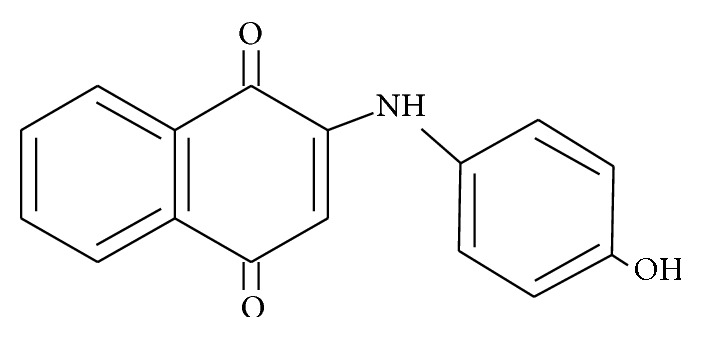
Chemical structure of 2-[(4-hydroxyphenyl)amino]-1,4-naphthoquinone (**Q7**).

The following drugs were used in this study: L-phenylephrine hydrochloride (PE; Sigma-Aldrich, St. Louis, Mo), acetylcholine chloride (ACh; Sigma-Aldrich, Munich, Germany), sodium nitroprusside (SNP; Merck, Darmstadt, Germany), barium chloride dihydrate (BaCl_2_; Sigma-Aldrich, St. Louis, MO), sulfanilamide (Sigma-Aldrich, St. Louis, MO), N-(1-naphthyl)ethylenediamine (NED; Sigma-Aldrich, St. Louis, MO), tetramethoxypropane (Sigma-Aldrich, St. Louis, Mo), thiobarbituric acid (Merck, Darmstadt, Germany), butylated hydroxytoluene (Merck, Darmstadt, Germany), N_w_-nitro-L-arginine methyl ester (L-NAME; Sigma-Aldrich, St. Louis, MO), and BAPTA-AM (Invitrogen, USA). Drugs were dissolved in distilled deionized water. The solutions in Krebs-Ringer bicarbonate (KRB) were freshly prepared before each experiment.  Figure 1 shows chemical structure of 2-[(4-hydroxyphenyl)amino]-1,4-naphthoquinone (**Q7**; 265.07 g/mol); it was synthesized by amination of 1,4-naphthoquinone with 4-hydroxyphenylamine, under aerobic conditions, using CeCl_3_·7H_2_O as the Lewis acid catalyst as previously reported [35].

### 2.11. Statistical Analysis

Values are expressed as mean ± standard error of the mean; *n* denotes the number of animals studied. One- or two-way analysis of variance (ANOVA) was carried out to detect significant differences, followed by Bonferroni post-tests to compare all groups. A *p* value of <0.05 was considered statistically significant.

## 3. Results

### 3.1. Cytotoxicity of** Q7** on Vascular Endothelial Cell Line and Vascular Smooth Muscle

**Figure 2:**
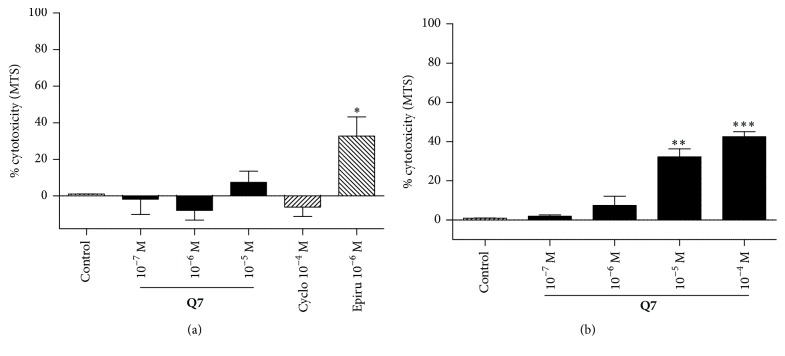
**Q7** does not induce cell death. The results show that** Q7** cytotoxicity was low or negligible on a vascular endothelial cell or vascular smooth muscle cell line, respectively.** Q7** cytoxicity was determined by MTS assay. Human umbilical vein endothelial cells (HUVEC) (a) and A7r5 vascular smooth cell line (b) were incubated in the absence or in the presence of** Q7** (10^−7^ to 10^−4^ M) for 48 h. Cyclophosphamide (Cyclo; 10^−4^ M) was used as negative control and epirubicin (Epirub; 10^−5^ M) as positive control. The cells were seeded at 50% density. Data are the average ± SEM of 4 independent experiments; ^*∗*^ *p* < 0.05; ^*∗∗*^ *p*; ^*∗∗∗*^ *p* < 0.001 versus control.

Because oxidative stress is associated with cell death, the effect of** Q7** on the viability of HUVEC and the vascular smooth muscle cell line A7r5 was assessed (Figure 2). After 48 h of incubation, the cytotoxicity in presence of 10^−5 ^M** Q7** did not significantly increase in endothelial cells (1.00 ± 0.05% in control versus 7.39 ± 6.14% with 10^−5^ M** Q7**) and increased significantly in vascular smooth muscle cells (0.97 ± 0.03% in control versus 32.20 ± 4.04% with 10^−5 ^M** Q7**; *p* < 0.01). As expected, no significant cytotoxicity was observed with the chemotherapy cyclophosphamide (10^−4 ^M), which is activated only after metabolism in the liver and thus is inactive in cell culture.

### 3.2. **Q7** Induces Oxidative Stress in Rat Aorta and Decreases Endothelial NO Production in Rat Aorta

**Figure 3:**
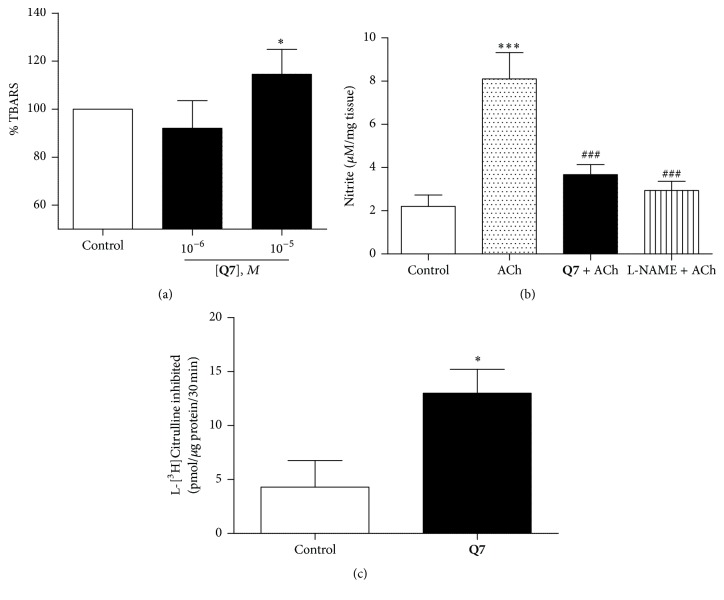
**Q7** produces oxidation of lipids and* decreases endothelial NO*. Depicting oxidative stress revealed by** Q7**-mediated lipid oxidation and a change of endothelial NO release in rat aorta tissue. The rat aorta homogenate determination of TBARS in presence of** Q7** (10^−6^ and 10^−5^ M) was measured as % with respect to control (a). Significant differences were found between 10^−5^ M** Q7** versus control or 10^−6^ M of** Q7** (^*∗*^ *p* < 0.05). The production of NO by a segment of the rat aorta was measured by the accumulation of nitrite using Griess reaction method (b): the aortic rings were incubated with vehicle (control), 10^−5^ M ACh, 10^−5^ M** Q7** plus 10^−5^ M ACh, and 10^−5^ M ACh plus 10^−4^ M L-NAME. Data are the average ± SEM of 5 independent experiments. ^*∗∗∗*^ *p* < 0.001 versus control; ^###^ *p* < 0.001 versus ACh; effect of** Q7** on eNOS activity and production of endothelial NO (c). The production of NO was determined indirectly by the formation of L-citrulline in the reaction catalyzed by eNOS in HUVEC. The results are mean standard error of the mean. Asterisk indicates statistically significant differences compared to the control (^*∗*^ *p* < 0.05; *n* = 3).

We conducted experiments to evaluate oxidative stress revealed by** Q7**-mediated lipid oxidation in rat aorta tissue. TBARS assay was used as an index of lipid peroxidation. As shown in Figure 3(a),** Q7** (10^−5^ M) increased the formation of TBARS by 114 ± 5% as compared to control (vehicle).

To unravel the modulatory effects on mechanisms involved in vasodilation, NO formation in rat aorta was assessed by measuring the production of nitrites using the Griess reaction. The aortic rings were incubated with vehicle, ACh (10^−5 ^M), and** Q7** (10^−5 ^M) + ACh (10^−5 ^M) for 30 min.  Figure 3(b) shows that the preincubation with** Q7** significantly decreased the formation of endothelial NO (8.1 ± 1.2 nitrite *μ*M/mg tissue with ACh versus 3.7 ± 0.5 nitrite *μ*M/mg tissue with** Q7 **+ ACh, *p* < 0.01). The preincubation with L-NAME also decreased significantly the production of nitrites (2.9 ± 0.4 nitrite *μ*M/mg tissue with L-NAME + ACh, *p* < 0.01).

We measured eNOS activity in presence of** Q7** by the formation of L-citrulline in the reaction catalyzed by eNOS in HUVEC (Figure 3(c)). The concentration of L-citrulline formed is directly proportional to the NO concentration formed and eNOS activity. We showed that** Q7** (10^−5 ^M) increased the concentration of L-citrulline in HUVEC (4.29 ± 2.46 pmol/*μ*g protein control, 12.99 ± 2.22 pmol/*μ*g protein with 10^−5 ^M** Q7**; *p* < 0.05).

**Figure 4:**
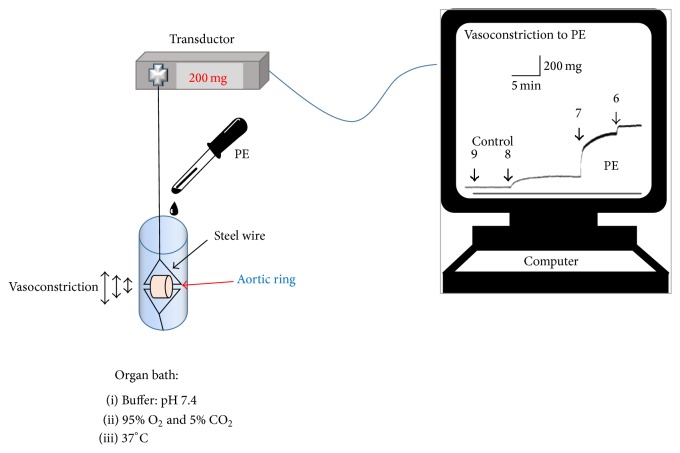
Vascular reactivity experimental setup. Aortic ring was mounted on two steel wires; the upper one was attached to an isometric transducer. An equilibration period and stabilization with KCl were allowed, and the tissue was washed two times with fresh KRB. Then, we performed the protocol of the experiment; we added different concentrations of PE, which caused vasoconstriction of aortic ring. To finish, data were registered in the computer. The increase of the trace of the screen indicates vasoconstriction to PE, while the decrease of the trace indicates vasodilatation of the aortic ring.

**Figure 5:**
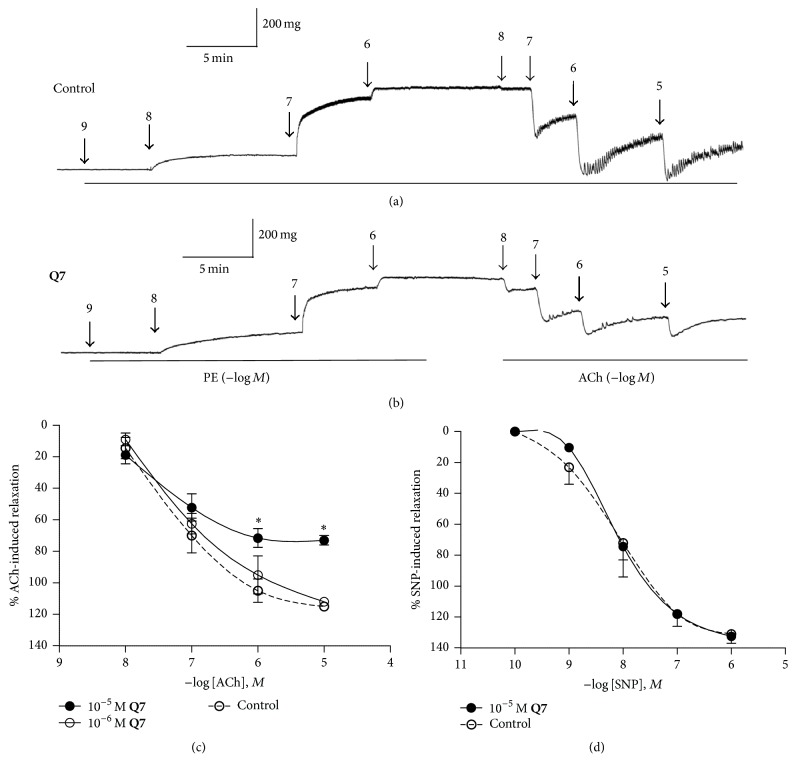
**Q7** impairs ACh-induced vasodilation in an NO-dependent mechanism. Original trace showing the time course of the concentration–response curves to PE (10^−9^–10^−6^ M) and ACh (10^−8^–10^−5^ M) in intact aortic rings of rats: control (a) and in the presence of 10^−5^ M** Q7** for 30 min (b). After an equilibration period and before PE, the aortic rings were stabilized by two successive near-maximal contractions with 6 × 10^−2^ M KCl. ACh-response curves in endothelium-intact rat aorta in the presence or absence (control) of** Q7** (10^−6^ M, 10^−5^ M) (c). Arteries were preconstricted with 10^−6^ M PE. SNP-response curves in rat aorta in the presence or absence (control) of 10^−5^ M** Q7** (d). Data are the average ± SEM of 4-5 independent experiments. ^*∗*^ *p* < 0.05 versus control.

To test whether decreased or increased NO might have an effect on vasodilation, we tested the modulatory effect of** Q7** on ACh-induced vasodilation and PE-induced vasoconstriction (Figures 4 and 5).

### 3.3. Endothelial Vasodilation of Rat Aortic Rings Induced by ACh Is Impaired by** Q7**

To confirm that** Q7** impairs endothelial dependent vasodilation via a NO pathway, further experiments were conducted in PE (10^−6 ^M) precontracted aortas and doses of either ACh (10^−8^–10^−5^ M; Figures 5(a)–5(c)) or SNP (10^−10^–10^−6 ^M; Figure 5(d)) were increased. As shown in Figures 5(b) and 5(c), preincubation for 30 min with** Q7** (10^−5 ^M) results in a significant decrease of ACh-mediated vasodilation of intact aortic rings: 115 ± 2% control versus 73 ± 3% with 10^−5 ^M** Q7** (10^−5 ^M ACh; *p* < 0.05). This finding suggests that** Q7** decreases the endothelial nitric oxide, ACh-induced, which agrees with decreased production of nitrites observed in previous experiments. Nevertheless, by applying the same experimental protocol and using a NO-donor compound, namely, SNP, a vasodilation of 100% was observed even in the presence of 10^−5 ^M** Q7** (Figure 5(d)). This indicates that the reduction of vasodilation can be overcome by NO; therefore,** Q7** only impairs the endothelial response and there was no response of vascular smooth muscle.

In the next experiments, we studied if the decreased vasodilation should be accompanied by reduced intracellular calcium in precontracted aorta with PE.

### 3.4. Effect of** Q7** on the Calcium Homeostasis in A7r5 Cells

**Figure 6:**
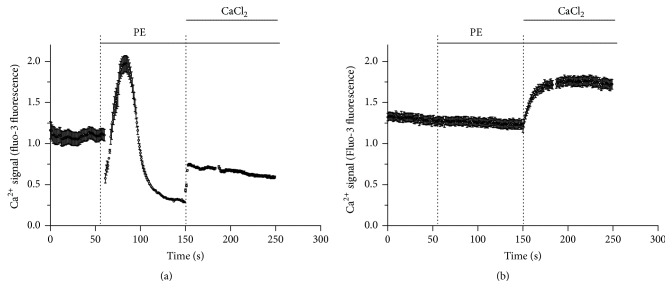
Quinone effect on intracellular Ca^2+^ levels in A7r5 cells.** Q7** blunted the release of calcium from intracellular stores, but not the influx of calcium from extracellular space. Representative plots of relative changes in Ca^2+^ signal (fluo-3 fluorescence) over time on A7r5 cells in calcium-free medium with 10^−6^ M phenylephrine (PE) in absence of** Q7** (control) (a) or presence of 10^−5^ M** Q7** (b). Data are the average ± SEM (*n* = 3).

To investigate if the contractile response of PE in presence of** Q7** could be modulated by calcium released from intracellular stores, further experiments were conducted with PE in A7r5 cells in a calcium-free medium.  Figure 6 shows that this situation, 10^−5 ^M** Q7,** drastically blunted the release of calcium from intracellular stores in response to 10^−6 ^M PE, but it however, did not blunt the increase of intracellular calcium when 1 mM CaCl_2_ was added to extracellular space.

In order to gain insight into potential role of** Q7** on vascular reactivity, we repeat a similar protocol in response to PE in aortic rings.

### 3.5. Effect of** Q7** on Aortic Rings Vasoconstriction Induced by PE

**Figure 7:**
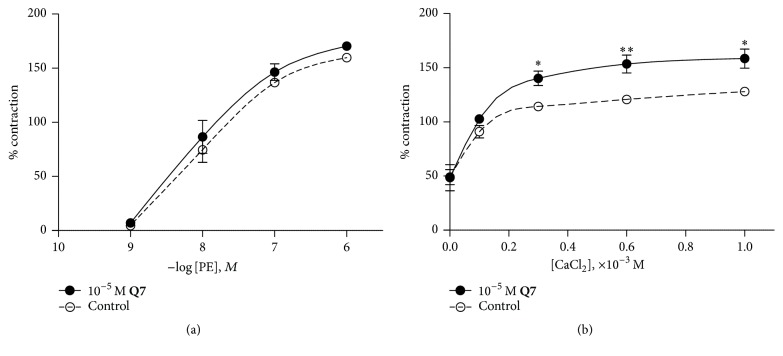
**Q7** increases PE-dependent vasoconstriction in a Ca^2+^-dependent manner. PE-response curves in endothelium-intact rat aorta in the presence or absence (control) of 10^−5^ M** Q7** (a). The vascular tissue was preincubated in a KRB buffer without calcium for 10 min before 10^−6^ M PE was added; and then, the CaCl_2_ (0.1, 0.3, 0.6, and 1.0 × 10^−3^ M) was added to the bath (b). Data are the average ± SEM of 4-5 independent experiments. ^*∗*^ *p* < 0.05 and ^*∗∗*^ *p* < 0.01 versus control.

The effect of** Q7** preincubation on the contractility response to PE was explored. As shown in Figure 7(a),** Q7** at 10^−5 ^M did not potentiate the contractile effect induced by PE when tested in the presence of normal KRB (1.3 × 10^−3^ M extracellular calcium). However,** Q7** enhanced the contractile response to PE when calcium was added to extracellular space in a calcium-free medium. No change in the basal tension of aortic rings was observed by** Q7** *per se*. We investigated if the contraction induced by PE in presence of** Q7** could be modulated by calcium inflow from the extracellular space. Thus, we added increasing concentrations of CaCl_2_ to a calcium-free medium.  Figure 7(b) shows that in the presence of 10^−3 ^M CaCl_2_,** Q7** (10^−5 ^M) significantly increased the contractile response to PE (10^−6 ^M) from 128 ± 1% control to 158 ± 9% (*p* < 0.05). Percentages were determined with respect to submaximal contraction with 6 × 10^−2^ M KCl. This data suggests that** Q7** increased calcium influx thus enhancing PE-induced vasoconstriction.

### 3.6. Role of Potassium Channels in the Vascular Response to** Q7** in Rat Aortic Rings

**Figure 8:**
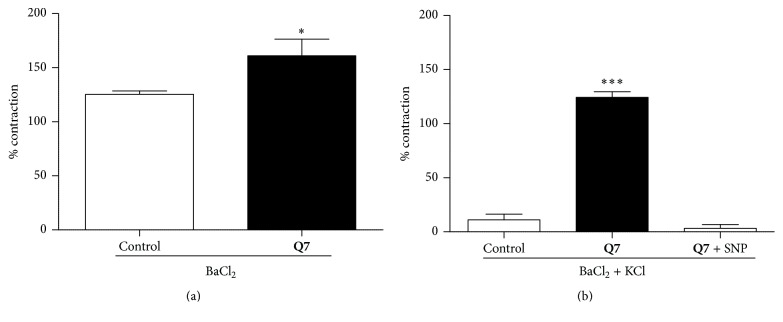
Acute effect of** Q7** on potassium channels in rat aorta.** Q7** significantly decreases the vascular contractile response involved potassium channels. The contraction with 10^−3^ M BaCl_2_ (a) and effect of 10^−2^ M KCl (b) in rat aorta precontracted with 10^−3^ M BaCl_2_ are shown. Open bar (control, vehicle), black bar (10^−5^ M** Q7**), and striped bar (10^−5^ M** Q7** + 10^−8^ M SNP). Percentages of contraction with BaCl_2_ were determined with respect to submaximal contraction with 6 × 10^−2^ M KCl (a), and the percentages of contraction in presence of 10^−2^ M KCl were determined with respect to maximal contraction with 10^−3^ M BaCl_2_ (b). Data are the average ± SEM of 4-5 independent experiments. ^*∗*^ *p* < 0.05 and ^*∗∗∗*^ *p* < 0.001 versus control.

The putative role of potassium channels on vascular contractile response was further studied. For this purpose, BaCl_2_ was used, as it increases vasoconstriction by blocking voltage-dependent Ca^2+^-sensitive K^+^ channels.  Figure 8(a) shows that preincubation with** Q7** significantly enhanced the contractile response to BaCl_2_ (10^−3 ^M): 125 ± 3% control versus 161 ± 15% with 10^−5 ^M** Q7** (*p* < 0.05). Interestingly, low concentrations of KCl caused repolarization and decreased the vasoconstriction, but, in the presence of** Q7**, 10^−2 ^M KCl did not decrease the vasoconstriction provoked by BaCl_2_. Only when a NO donor (10^−8 ^M SNP) was added to the preparation, the aortic rings were able to recover 100% of its vasodilation, reaching values similar to control conditions (Figure 8(b)). These data suggest that potassium channels are required for the modulatory effect of** Q7** on vascular contractile response and in consequence, the compound might affect the membrane potential.

## 4. Discussion

The modulation of blood flow through the vascular endothelium influences the progression of several pathologies [36, 37]. Although studies have shown that quinone related compounds can impair vasodilation by endothelial dysfunction [3, 4, 38], the effect of quinones on endothelial function at low concentrations is not well characterized.

We found that noncytotoxic doses of** Q7** induces oxidative stress and reduces the formation of endothelial NO in rat aorta, thus decreasing endothelial NO-dependent vasodilation. Our findings are consistent with a probable mechanism in which oxidative stress and decrease of NO may partially block potassium channels, thus depolarizing the cell membrane leading in consequence to the opening of calcium channels, increased calcium influx, and thus increasing calcium-dependent vasoconstriction.

Herein, we show that** Q7** cytotoxicity after 48 h on vascular endothelial cell was negligible and on vascular smooth muscle cell was low. Considering that the incubation time in experiments conducted to determine vascular reactivity and oxidative stress in rat aortic rings lasted 30 min, it appears unlikely that such effects are the consequence of** Q7** cytotoxicity.

In the present study, we observed that** Q7** significantly increased lipid peroxidation in rat aorta homogenates, as shown by the enhanced TBARS formation. This finding was attributed to the capacity of** Q7** to produce oxidative stress by increasing of ROS. In previous studies, we showed that TBARS increased in calf-thymus DNA treated with** Q7 **or juglone [39]. Free radicals can attack DNA at C4 of deoxyribose generating products as propenal, which react with 2-thiobarbituric acid and produce the TBARS formation [40]. In both T24 and MCF-7 cells, we found that** Q7** provoked elevated levels of intracellular ROS [23, 25, 35, 39].

Oxidative stress produced by** Q7** is mainly due to ROS generation through a redox-cycling mechanism as described for other quinones [12]. This might affect critically NO levels in the rat aorta.** Q7** increased the formation of L-citrulline in HUVEC, compatible with increased NO generation and eNOS activity [41]. This paradoxical result can be explained in part because the ROS produced by redox-cycling of** Q7** react rapidly with generated NO, leading to a reduction of its level [38]. Quinones (i.e., doxorubicin, menadione) increase the generation of ROS (anion superoxide) leading to scavenging of NO, in agreement with previously described results [42–44]. In fact, we now found that** Q7** significantly reduced the ACh-mediated NO formation.

Accordingly, ACh-mediated endothelial vasodilation was significantly reduced by** Q7**, but it was recovered when a NO donor (SNP) was added into intact rat aorta preparations. Therefore, endothelial dysfunction caused by** Q7** may be explained by a decrease in the bioavailability of endothelial NO.

Oxidative stress is associated with disruption of intracellular calcium homoeostasis [45]. In fact,** Q7** blunted the release of calcium from intracellular stores in response to PE on vascular smooth muscle cell (A7r5 cells) in a free-calcium medium. Similar results we observed in cardiac fibroblasts preincubated with** Q7** in a free-calcium medium in presence of angiotensin II (data not shown). These findings suggest that oxidative stress induced by** Q7** blocked the release of intracellular calcium or caused calcium leakage from intracellular stores to extracellular space. This is in agreement with the inhibitory effect of quinone-related compounds (menadione) on the release of calcium from intracellular stores [46], by inhibition of sarcoplasmic calcium ATPase [47]. It is possible that the endoplasmic reticulum emptying of calcium by** Q7** enhanced the contractile response to PE of aortic rings when calcium was provided extracellularly. We found that** Q7** did not decrease the influx of calcium from extracellular space.

We have obtained preliminary data suggesting that** Q7** may also inhibit potassium channels in neurons. Our results show an increase in action potential firing that is accompanied by increased input resistance (data no shown), an effect that could be mediated by blockade of hyperpolarization-activated cyclic nucleotide gated (HCN) channels, that carry the Ih current [48]. However, more experiments are needed to confirm the participation of different ion channels in this.

To study a putative role of potassium channels in NO-dependent vasodilation, we use BaCl_2_. Barium blocks inward rectifying potassium channels such as ATP-sensitive K^+^ channels at submillimolar concentrations [49, 50] and voltage-dependent Ca^2+^-sensitive K^+^ channels at millimolar concentrations [29, 30]. The result of the blockage of potassium channels by barium induced contraction in aortic rings. In the presence of** Q7**, the addition of KCl (10^−2 ^M) was unable to provoke a total vasodilation in rat aorta preconstricted with BaCl_2_ (10^−3 ^M), an effect which was however obtained at 100% by adding SNP (10^−8 ^M). The increase of KCl over 2 × 10^−2^ M caused repolarization and vasodilation, because the membrane potential moves towards the new K^+^ equilibrium potential, and voltage-dependent Ca^2+^-sensitive K^+^ channels are again opened [29, 41, 51]. These findings may be explained by previous studies, showing that juglone (5-hydroxy-1,4-naphthoquinone) partially blocks voltage-dependent potassium channels, causing depolarization of the membrane [52].

These results agree with the idea that vasodilation induced by endothelial NO is due, at least in part, to a repolarization of the plasma membrane by opening voltage-dependent Ca^2+^-sensitive K^+^ channels [53–55] and closing of L-type Ca^2+^ channels [56], decreasing in this way the calcium influx [57, 58].

In conclusion, oxidative stress induced by** Q7** decreases endothelial vasodilation, a process likely accompanied by decreased NO bioavailability and partial downstream blockade of potassium channels and membrane depolarization, followed by enhanced influx of calcium [59]. These findings could have interesting and potential clinical effects for a number of pathologies such as inflammatory disorders, diabetic blindness, age-related muscular degeneration, psoriasis, cardiovascular and autoimmune diseases, and cancer [60].
